# Multiplex PCR-based next generation sequencing as a novel, targeted and accurate molecular approach for periprosthetic joint infection diagnosis

**DOI:** 10.3389/fmicb.2023.1181348

**Published:** 2023-05-18

**Authors:** Changyu Huang, Ying Huang, Ziwen Wang, Yiming Lin, Yongbin Li, Yang Chen, Xiaoqing Chen, Chaofan Zhang, Wenbo Li, Wenming Zhang, Xinyu Fang, Zida Huang

**Affiliations:** ^1^Department of Orthopaedic Surgery, National Regional Medical Center, Binhai Campus of the First Affiliated Hospital, Fujian Medical University, Fuzhou, China; ^2^Department of Orthopedic Surgery, The First Affiliated Hospital of Fujian Medical University, Fuzhou, China; ^3^Fujian Provincial Institute of Orthopedics, The First Affiliated Hospital, Fujian Medical University, Fuzhou, China; ^4^Fujian Medical University, Fuzhou, China; ^5^Department of Orthopedic Surgery, Quanzhou First Hospital Affiliated to Fujian Medical University, Quanzhou, China

**Keywords:** periprosthetic joint infection, targeted next-generation sequencing, metagenomic next-generation sequencing, culture, diagnostics

## Abstract

**Objectives:**

Periprosthetic joint infection (PJI) diagnosis remains challenging, and the identification of the causative microorganism is, by far, the most important aspect. Here, we use multiple PCR-based targeted next-generation sequencing (tNGS) to detect pathogens in PJI. To explore 1. the ability of targeted next-generation sequencing (tNGS) to detect pathogens in PJI; 2. the consistency of tNGS, metagenomic NGS (mNGS), and culture results; and 3. the ability of tNGS to detect drug resistance genes in PJI.

**Methods:**

PJI was diagnosed according to the Musculoskeletal Infection Society (MSIS) criteria. The microorganisms were detected by culture, mNGS and tNGS to compare the diagnostic effectiveness of the three methods for PJI and to compare their consistency in detecting microorganisms. Drug resistance genes were detected using tNGS. The costs and turnaround times of mNGS and tNGS were compared.

**Results:**

Forty-three patients with PJI, 21 patients without PJI and 10 negative control cases were included. The culture, tNGS, and mNGS sensitivities for PJI diagnosis were 74.41%, 88.37%, and 93.02%, respectively, with no significant differences. The specificities were 90.48%, 95.24%, and 95.24%, respectively, with no significant differences. tNGS detected drug resistance genes in 37.5% of culture-positive PJIs. tNGS was superior to mNGS for turnaround time (14.5 h vs. 28 h) and cost ($150 vs. $260).

**Conclusions:**

tNGS can effectively identify PJI pathogens and may provide drug resistance information, while tNGS is superior to mNGS regarding cost and turnaround time. A multidisciplinary, multi-technology based algorithm to diagnose PJI is appropriate.

**Highlights:**

1. 298 microorganisms and 86 drug resistance genes were included in the tNGS panel.
2. Diagnostic efficacy of tNGS is not inferior to that of commonly used indicators.
3. tNGS is superior to mNGS in cost and turnaround time.

**Graphical abstract:**
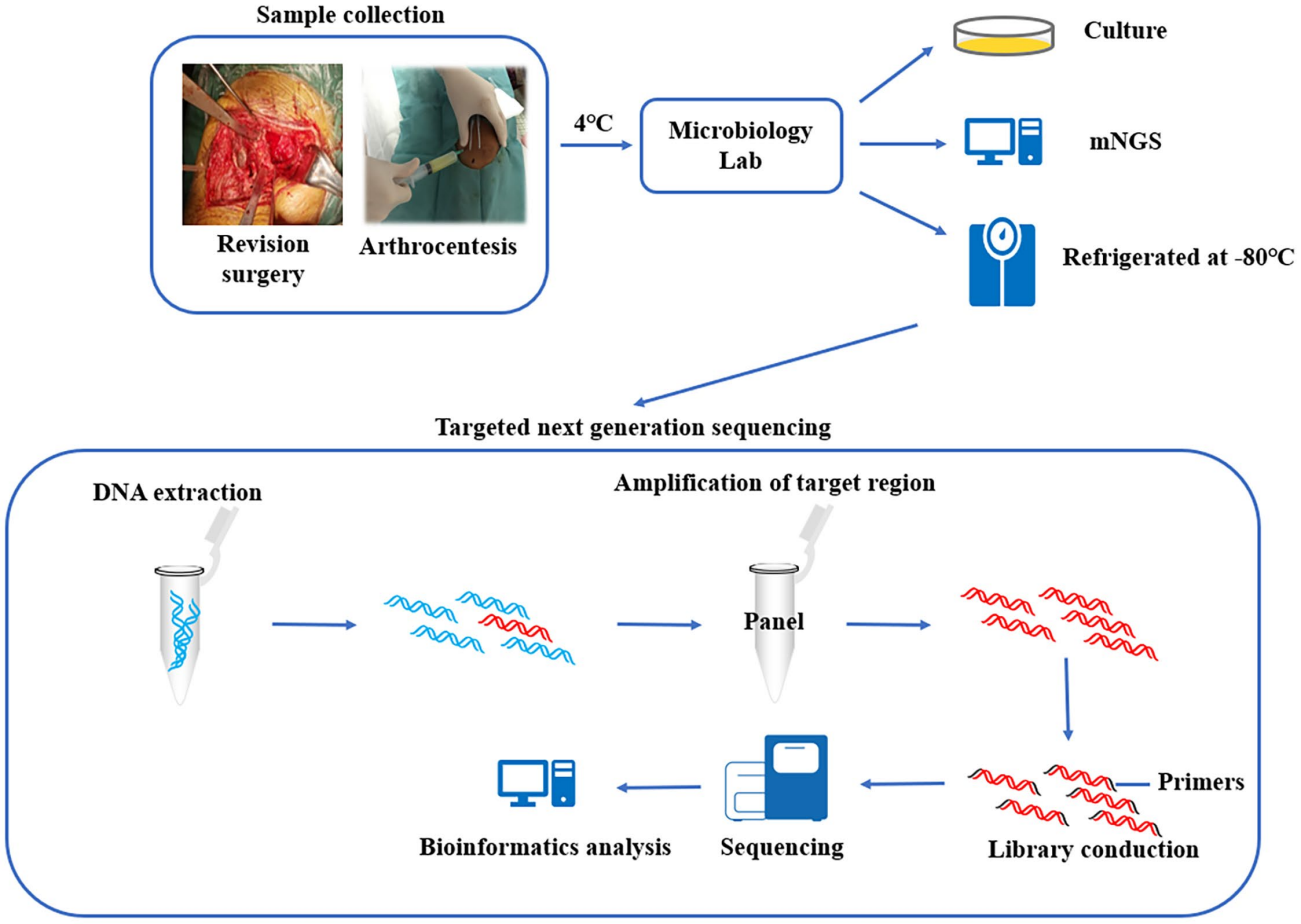
1. Synovial fluid were tested for pathogenic bacteria using culture and mNGS, and the remaining specimens were preserved in a biobank. 2. The preserved specimens were tested using multiplex PCR-based next generation sequencing. 3. Compare the diagnostic efficacy of the three methods for PJI.

## Introduction

Periprosthetic joint infection (PJI) diagnosis remains challenging. Diagnostic criteria such as those developed by the American Academy of Orthopaedic Surgeons and Musculoskeletal Infection Society (MSIS) provide good support for the diagnosis of PJI, and among these criteria, culture is considered the most critical aspect, which provides information not only on pathogenic microorganisms but also on antibiotic resistance (Workgroup Convened by the Musculoskeletal Infection Society, 2011; Parvizi et al., 2013, 2018; Tubb et al., 2020). However, biofilm formation, prior antibiotic use and fastidious pathogens contribute to the low sensitivity of conventional culture (Stoodley et al., 2011; Wouthuyzen-Bakker et al., 2017), and despite strategies such as sonication and other optimized culture protocols being used to improve detection rates (Trampuz et al., 2007; Fang et al., 2021), the rate of culture-negative PJI (CN-PJI) still ranges from 7.0 to 42.1% (Yoon et al., 2017).

In contrast with time-consuming and low-sensitivity culture, nucleic acid detection technology can detect pathogenic bacteria without culture, and based on the principle of “no preset target,” the detection sensitivity of these techniques has been significantly improved. 16S ribosomal RNA gene polymerase chain reaction (PCR) can be used to detect most bacteria, but it cannot identify fungi or polymicrobial infections and cannot quantitatively distinguish contaminating bacteria (Huang et al., 2018). Metagenomic next-generation sequencing (mNGS), also known as metagenomic shotgun NGS, can overcome the shortcomings of 16S rRNA PCR and detect all known/unknown pathogens that cause bone and joint infection, including fungi, bacteria, mycoplasma, parasites, etc., and can provide multidimensional quantitative test results for the identification of polymicrobial infection and contaminating bacteria (McCombie et al., 2019). This has led to effective use of mNGS in PJI. Street et al. applied mNGS to identify pathogens in PJI with a sensitivity of 93% (Street et al., 2017). Thoendel et al. applied mNGS to detect pathogens in 94.8% of culture-positive PJIs (CP-PJIs) and identified new potential pathogens in 43.9% of CN-PJIs (Thoendel et al., 2018). However, even with continued technological advances, the unbiased sampling of mNGS makes it challenging to exclude human-derived genes from samples, and this challenge leads to waste of detection resources and a decrease in sensitivity (Chiu and Miller, 2019). Moreover, human-derived genes result in relatively low reads of pathogen genomes, which makes it difficult for mNGS to detect drug resistance genes to inform the use of sensitive antibiotics.

Multiple PCR-based targeted NGS (tNGS) is performed by making a panel of the specific sequences of prescreened pathogens, amplifying the target genes, obtaining information about the enriched nucleic acids through a high-throughput sequencing platform, and then analyzing the results by bioinformatics to identify pathogens (Graphical abstract). Based on the predefined panel, tNGS can eliminate the interference of human-derived genes. In theory, the sensitivity of diagnosis can be high as long as the coverage of pathogens is sufficiently broad. This technique has now been used for pathogen identification in cases of pulmonary infections, brain infections, and *Mycobacterium* infections, as well as for the detection of drug resistance genes, and has been shown to have potential for clinical application (Chao et al., 2020; Gao et al., 2021; Huang et al., 2021; Li et al., 2021). Here, we design a panel of specific sequences of 298 pathogens as well as 86 drug resistance genes and applied them to the diagnosis of PJI using tNGS.

The objectives of this study are as follows: 1. to evaluate the ability of tNGS to detect pathogens from PJI; 2. to verify the consistency of the results between tNGS and mNGS and culture; and 3. to explore the ability of tNGS to detect drug resistance.

## Materials and methods

This study was approved by the Ethics Committee of our institution (MTAC, ECFAH of FMU [2015] 084-2, 2018 [026]). The synovial fluid used for tNGS detection in this study were obtained from our clinical biobank, and were collected and retained with the informed consent of the patients. Synovial fluid collected between April 2020 and September 2022 were included. Inclusion criteria included the following: 1. patients undergoing joint revision surgery; 2. those who had mNGS testing during treatment and had complete microbiological data. Incomplete clinical records and potentially contaminated samples were criteria for exclusion. Clinical data of patients were obtained from the electronic medical records. The MSIS criteria was used as the gold standard to diagnose PJI (Workgroup Convened by the Musculoskeletal Infection Society, 2011). In addition, 10 patients (osteoarthritis) undergoing primary total hip/knee arthroplasty without a history of inflammatory arthritis, joint infection, or prior surgery were recruited as negative controls. The cost and turnaround time of tNGS and mNGS were defined as the cost and time incurred from the beginning of DNA extraction to the time when the result is obtained. The antibiotic regimen of all patients was determined by a multidisciplinary team based on a combination of culture and mNGS results.

### Sample collection

Synovial fluid samples were obtained from patients during revision surgery, in short, the synovial fluid was aspirated with a syringe before the joint capsule was incised at the time of surgery, or if sufficient joint fluid cannot be obtained, the joint capsule was incised and then aspirated under direct vision. All surgeries were performed by the same surgical team. The collected samples were immediately transferred to the microbiology department for further processing, and the specimens were kept at 4°C during transport. The collected samples were used for conventional microbial culture and mNGS. Finally, the remaining synovial fluid (raw specimen, unprocessed) was packaged in DNase-free and RNase-free sterile cryogenic vials and stored at −80°C (Graphical abstract). Synovial fluid was retained for 12.74 months (range: 1–29 months) and tested by tNGS in September 2022.

### Culture procedure

Intraoperative synovial fluid specimens were injected directly into commercial culture flasks (Becton-Dickinson GmbH, Heidelberg, Germany) within 2 h of acquisition and cultured in a specialized incubator. All the culture time was more than 7 days. Bacterial identification and drug sensitivity testing were performed using an IVD MALDI Biotyper system (Microflex LT/SH, Germany) and a Vitek II system (BioMérieux, USA).

### mNGS process

The mNGS process was performed as described previously (Huang et al., 2020a). Briefly, the extracted total genomic DNA was processed to generate fragments. DNA libraries were constructed by end repair, specific adaptor ligation, purification, PCR, and cyclization reactions to generate single-stranded DNA circles. The quantitative library was sequenced on the BGISEQ-2000 platform (BGI-Wuhan, Wuhan, China) for 50 bp single-end sequencing. Finally, the raw sequencing data were analyzed using the bioinformatics pipeline (containing 32 drug resistance genes) developed by BGI.

### tNGS process

This method included 298 pathogens (Supplementary Material S1) potentially associated with PJI in the panel, based on the official Chinese published National CDC Catalogue of Human Pathogenic Microorganisms, previous data from our institution, and available literature reports. The pathogens included fungi (*n* = 64), gram-positive bacteria (*n* = 120), gram-negative bacteria (*n* = 82), bacterial genera (*n* = 16), and others (*n* = 16). Eighty-six resistance genes for 13 resistance phenotypes (Supplementary Material S2) were also included in the panel. The production and testing of the panel was performed in-house by Shanghai Pathogeno Medical Technology Co., Ltd.

The method of total genome DNA extraction was consistent with that of mNGS. The DNA products were used as templates for multiplex PCR amplification using the abovementioned panel. Then, sequencing linkers and barcode sequences for sample identification were added to obtain pathogen sequencing libraries. Library concentrations were quantified using a Qubit 4.0 fluorometer (Invitrogen), requiring that the amount of data that could be fractionated to each pathogen library was no less than 0.05 M reads, and then the libraries were mixed. The concentration of the mixed libraries was accurately quantified using a Qubit 4.0 fluorometer, and the library was denatured after dilution to a final concentration of 4 nM. High-throughput sequencing was performed using an Illumina MiSeq Reagent Nano Kit and the Illumina MiSeq sequencing platform, with an average data volume of 0.03 ~ 0.05 M reads per library and a sequencing read length of PE75. The raw data obtained were first analyzed by identifying the reads with the linker sequence, trimming the linker and the subsequent sequences, and filtering the low-quality data. The high-quality data were identified by primer sequences, and the reads with correct paired-end overlap were then compared with the pathogen sequences in RefSeq, GenBank, and other databases downloaded from NCBI to finally determine the pathogen species and content in the samples (Graphical abstract).

The interpretation of the mNGS and tNGS results was done by a multidisciplinary team (including infection physicians, microbiologists, orthopedists) based on the methods described in our previous study (Huang et al., 2020b).

### Statistical analysis

Variables conforming to a normal distribution are described by the mean ± standard deviation, and variables with a nonnormal distribution are described by the median and interquartile range (IQR). Statistical significance analysis was performed with the *t* test, the chi-square test, Fisher’s exact probability method or the Mann–Whitney test according to the characteristics of the variables. Statistical differences in the sensitivity and specificity of culture, tNGS, mNGS, CRP, ESR, SF-WBCs, and SF-PMNs % were analyzed using McNemar’s test. *p* values ≤0.05 were considered statistically significant. Statistical analysis was performed using SPSS 26.0 (IBM, Armonk, New York, USA).

## Results

### Demographic characteristics

Synovial fluid from 67 patients were included, and three were excluded: two samples suspected of contamination, and one patient had incomplete clinical data. The remaining 64 patients had a mean age of 63.60 ± 12.57 years, resulting in samples from 32 females and 34 hips and 30 knees. According to the MSIS criteria, there were 43 cases of PJI (including 11 CN-PJI cases and 32 CP-PJI cases) and 21 cases of non-PJI (Non-infectious arthroplasty failure, such as: prosthesis loosening). In addition, specimens from 10 patients with primary joint arthroplasty were included as negative controls. In the latest evaluation, there were no delayed-onset infections (>1 year) in either the non-PJI or negative control groups. Clinical and laboratory data for all cases are shown in Table 1.

**Table 1:** Clinical and laboratory characteristic of all cases.

Characteristic	Patients (*n* = 64)				Control (*n* = 10)	Value of *p* (Suspected-PJI vs. Control)
	Total	Non-PJI (*n* = 21)	PJI (*n* = 43)	Value of *p* (non-PJI vs. PJI)		
Age (yrs), $\bar{X}$ ± S	63.60 ± 12.57	63.66 ± 10.84	63.58 ± 13.46	0.50^b^	63.40 ± 8.80	0.21^b^
Female (n)	33	15	18	0.03^a^	5	>0.99^a^
BMI (kg/m^2^)	25.01 ± 1.69	24.64 ± 2.72	25.19 ± 2.68	0.84^b^	24.50 ± 2.42	0.40^b^
aCCI	2.0 (1.00,3.00)	2 (1.50,3.00)	2 (1.00,3.00)	0.51^c^	2 (1.00,2.25)	0.37^c^
Joint involved (n)						
Hip	34	13	21	0.97^a^	4	0.51^a^
Knee	30	8	22		6	
Surgical strategies						
One-stage	22	18	4	<0.01^a^		
Two-stage	40	3	37			
DAIR	2	0	2			
Interval between primary arthroplasty and revision (mths) $\bar{X}$ ± S		38.10 ± 26.48	43.30 ± 28.86	0.51^b^		
Antibiotics within 2 weeks prior to surgery (*n*)	16	4	12	0.47^a^	0	
Sinus (*n*)	17	1	16	<0.01^a^	0	
CRP (mg/L), median, IQR	15.1 (3.82,87.12)	3.81 (2.00,6.52)	39.6 (12.10,98.00)	<0.01^c^	2.98 (1.17,9.45)	<0.01^c^
ESR (mm/h), $\bar{X}\;$ ± S	55.79 ± 31.48	26.71 ± 14.02	65.76 ± 29.16	<0.01^b^	24.50 ± 4.35	<0.01^b^
SF-WBC (×10^6^/L), median, IQR	13,123 (984,85,337)	833 (419,1,154)	64,672 (12,055,108,964)	<0.01^c^		
SF-PMN% (%), median, IQR	82.0 (49.5,90.2)	45.9 (41.2,53.0)	87.6 (78.5,91.7)	<0.01^c^		
Positive periprosthetic tissue histopathology (*n*)	32	1	31	<0.01^a^		

PJI, Prosthetic joint infection; SF-WBC, Synovial fluid white blood cell; SF-PMN, Synovial fluid polymorphonuclear; BMI, Body mass index; aCCI, age-adjusted Charlson Comorbidity Index; DAIR, Debridement, Antibiotics and Implant Retention.

a Chi-squared.

b Independent-samples *t*-test.

c Mann-Whitney *U* test.

### Microorganisms detected by culture

**Figure 1:**
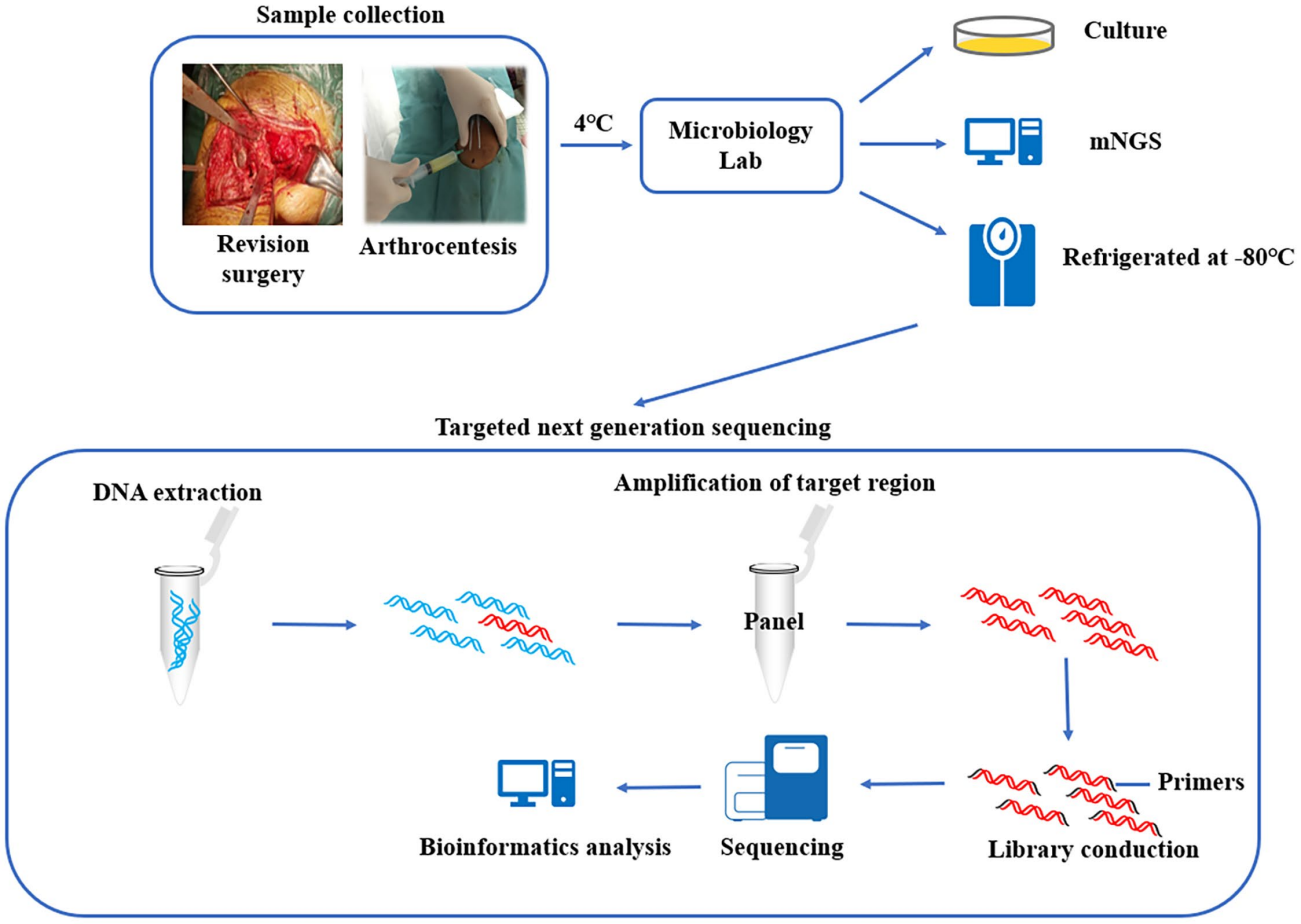
Results and comparison of culture, mNGS and tNGS. **(A)** Results of culture, mNGS and tNGS; (+): positive; (−): negative. (**B)** Culture and tNGS results match. **(C)** Culture and mNGS results match. **(D)** tNGS and mNGS results match.

For the 43 cases of PJI, microorganisms were isolated using culture in 32 cases, and a single pathogen was isolated in 30 of them (Figure 1A). Two patients were diagnosed as polymicrobial infection, and they yielded 2 strains (*Streptococcus anginosus + Staphylococcus aureus* and *Escherichia coli + Streptococcus agalactiae*) in different samples. The mNGS and tNGS results of these two patients with polymicrobial infection were consistent with the culture results. In 2 of the 21 non-PJI cases, microorganisms were isolated from a single sample and were considered false positives. In these two patients, antibiotic use was not prolonged postoperatively, and no reinfection was seen at follow-up (11 and 13 months of follow-up). No microorganisms were detected in the negative control group. Detailed culture result is shown in Table 2.

**Table 2:** Microbiology finding of culture, mNGS and tNGS in all cases.

Microorganism	Culture (*n*)	mNGS (*n*)	tNGS (*n*)
*Staphylococcus aureus*	15	14	17
*CoNS* ^a^	6	11	10
*Streptococcus* ^b^	7	8	8
Gram-negative bacilli^c^	3	4	3
*Pseudomonas* ^d^	3	1	4
*Enterococcus faecalis*	0	1	0
*Salmonella*	2	1	2
*Candida* ^e^	1	2	2
*Mycoplasma hominis*	0	1	1
Other organisms^f^	0	4	3
Total	37	47	45

mNGS, metagenomic next-generation sequencing; tNGS, targeted next-generation sequencing.

a Coagulase negative staphylococcus.

b Including *Streptococcus agalactiae*, *Streptococcus dysgalactiae*, *Streptococcus gallolyticus*, *Streptococcus anginosus*, *Streptococcus* spp.

c Including *Escherichia coli*, *Stenotrophomonas maltophilia*, *Enterobacter hormaechei*, *Afipia broomeae*.

d Including *Pseudomonas alcaligenes*, *Pseudomonas aeruginosa*, *Pseudomonas monteilii*, *Pseudomonas* spp.

e Including *Candida albicans*, *Candida parapsilosis*.

f Including *Corynebacterium striatum*, *Cutibacterium acnes*, *Finegoldia magna*, *Enterococcus casseliflavus*, *Neisseria* spp.

### Microorganisms detected by mNGS

Thirty of the 32 patients with CP-PJI tested positive by mNGS (93.8%) (Figure 1A). The mNGS results were in complete agreement (The test results were completely consistent) with the culture results for CP-PJI at the genus level in 81% of cases, at the species level in 52% of cases, in partial agreement (In addition to the same strain, there are some inconsistent results) in 13% of cases, and in complete disagreement (The test results are completely inconsistent) in 2 cases (2 cases with negative mNGS results) (Figure 1C). Among the 11 CN-PJI cases, mNGS detected microorganisms in 10 cases (90.9%), including *Staphylococcus aureus n* = 3, *Staphylococcus* spp. *n* = 1, *Streptococcus dysgalactiae n* = 1, *Candida albicans n* = 1, *Mycoplasma hominis n* = 1, *Cutibacterium acnes n* = 1, *Finegoldia magna n* = 1, and *Corynebacterium striatum n* = 1. Detailed mNGS result is shown in Table 2.

### Microorganisms detected by tNGS

Thirty of the 32 CP-PJI patients tested positive by tNGS (93.8%) (Figure 1A). The tNGS results were in complete agreement with the culture results for CP-PJI at the genus level in 78% of cases, at the species level in 62% of cases, in partial agreement in 9% of cases, and in complete disagreement in 4 cases (3 negative tNGS results and 1 inconsistent result) (Figure 1B). Among the 11 CN-PJI cases, tNGS detected microorganisms in 10 cases (90.9%), including *Staphylococcus aureus n* = 4, *Streptococcus dysgalactiae n* = 2, *Candida albicans n* = 1, *Mycoplasma hominis n* = 1, *Cutibacterium acnes n* = 1, and *Corynebacterium striatum n* = 1. Detailed tNGS result is shown in Table 2.

For PJI, the results of tNGS and mNGS were in complete agreement at the genus level in 72% of cases, at the species level in 60% of cases, in partial agreement in 12% of cases, and in complete disagreement in 8 unmatched cases (Figure 1D). Among the 8 unmatched cases, 4 cases had negative tNGS results, 3 cases had negative mNGS results, and the last case had both mNGS and culture results for *Pseudomonas alcaligenes*, while the tNGS result was positive for *Pseudomonas aeruginosa*. For the four patients that tested negative for tNGS, the results of mNGS and culture were positive for *Staphylococcus aureus n* = 2, *Streptococcus gallolyticus n* = 1, and *Finegoldia magna n* = 1; *Streptococcus gallolyticus* and *Finegoldia magna* were not included in our panel of tNGS.

### Antibiotic resistance detected by culture, mNGS and tNGS

Antibiotic resistance information was obtained using culture in 26 of 32 CP-PJI patients. mNGS detected the information of antibiotic resistance gene in only one sample (1/32), and the reading of specific pathogen in this patient was as high as 4,618. tNGS detected 11 different resistance genes corresponding to 8 resistance phenotypes in 37.5% of CP-PJI samples (12/32), and no resistance genes were detected in CN-PJI samples. tNGS detected resistance phenotypes consistent with drug susceptibility testing results in 75% (9/12) of cases. Penicillin resistance accounted for the highest proportion of drug resistance genes detected, with 58.3% of tNGS (7/12) and 53.8% of culture (14/26).

### Diagnostic efficacy of culture, mNGS and tNGS

According to the MSIS criteria, the sensitivity of culture, tNGS and mNGS for PJI was 74.41, 88.37 and 93.02%, respectively. The sensitivity of tNGS was not significantly different from those of culture and mNGS (*p* > 0.05). The specificities of culture, tNGS and mNGS were 90.5, 95.2 and 95.2%, respectively. The specificity of tNGS was not significantly different from those of culture and mNGS (*p* > 0.05). In addition, there were mostly no significant differences in sensitivity and specificity between tNGS and CRP, ESR, SF-WBC, and SF-PMN%. Finally, the sensitivity increased to 97.7%, and the specificity decreased to 85.7% when tNGS and culture were performed in parallel. The diagnostic efficacy of each indicator is listed in Table 3.

**Table 3:** Diagnostic efficiency of culture, mNGS and tNGS in PJI.

Variable	Non-PJI (*n* = 21)	PJI (*n* = 43)	Sensitivity	*p*-value^a^ (VS. tNGS)	Specificity	*p*-value^a^ (VS. tNGS)	PPA	NPA	Youden’s index	PPV	NPV	LR+	LR-
tNGS	1	38	88.4%	N/A	95.2%	N/A	0.88	0.95	0.84	97.4%	80%	18.56	0.12
Culture	2	32	74.4%	0.18	90.5%	>0.99	0.74	0.90	0.65	94.1%	63.3%	7.81	0.28
mNGS	1	40	93.0%	0.73	95.2%	>0.99	0.93	0.95	0.88	97.6%	87.0%	19.53	0.07
CRP (>10 mg/L)	2	33	76.7%	0.27	90.5%	>0.99	0.77	0.90	0.67	94.3%	65.5%	8.06	0.26
ESR (>30 mm/h)	9	36	83.7%	0.74	57.1%	<0.01	0.84	0.57	0.41	80%	63.2%	1.95	0.28
SF-WBC (>3,000 × 10^6^/L)	0	37	86.0%	>0.99	100%	N/A	0.86	1	0.86	100%	77.8%	N/A	0.14
SF-PMN% (>80%)	2	31	72.1%	0.12	90.5%	>0.99	0.72	0.90	0.63	93.9%	61.3%	7.57	0.31
tNGS + Culture	3	42	97.7%	0.13	85.7%	0.50	0.98	0.86	0.83	93.3%	94.7%	6.84	0.03
mNGS + Culture	3	42	97.7%	0.29	85.7%	0.63	0.98	0.86	0.83	93.3%	94.7%	6.84	0.03

Youden’ s index = sensitivity + specificity – 1; PJI, Prosthetic joint infection. PPA, Positive percent agreement. NPA, Negative percent agreement. LR+, Positive likelihood ratio; LR−, Negative likelihood ratio; NPV, Negative predictive value; PPV, Positive predictive value; mNGS, metagenomic next-generation sequencing; tNGS, targeted next-generation sequencing.

a McNemar’s test.

### Cost and turnaround time of tNGS and mNGS

**Figure 2:**
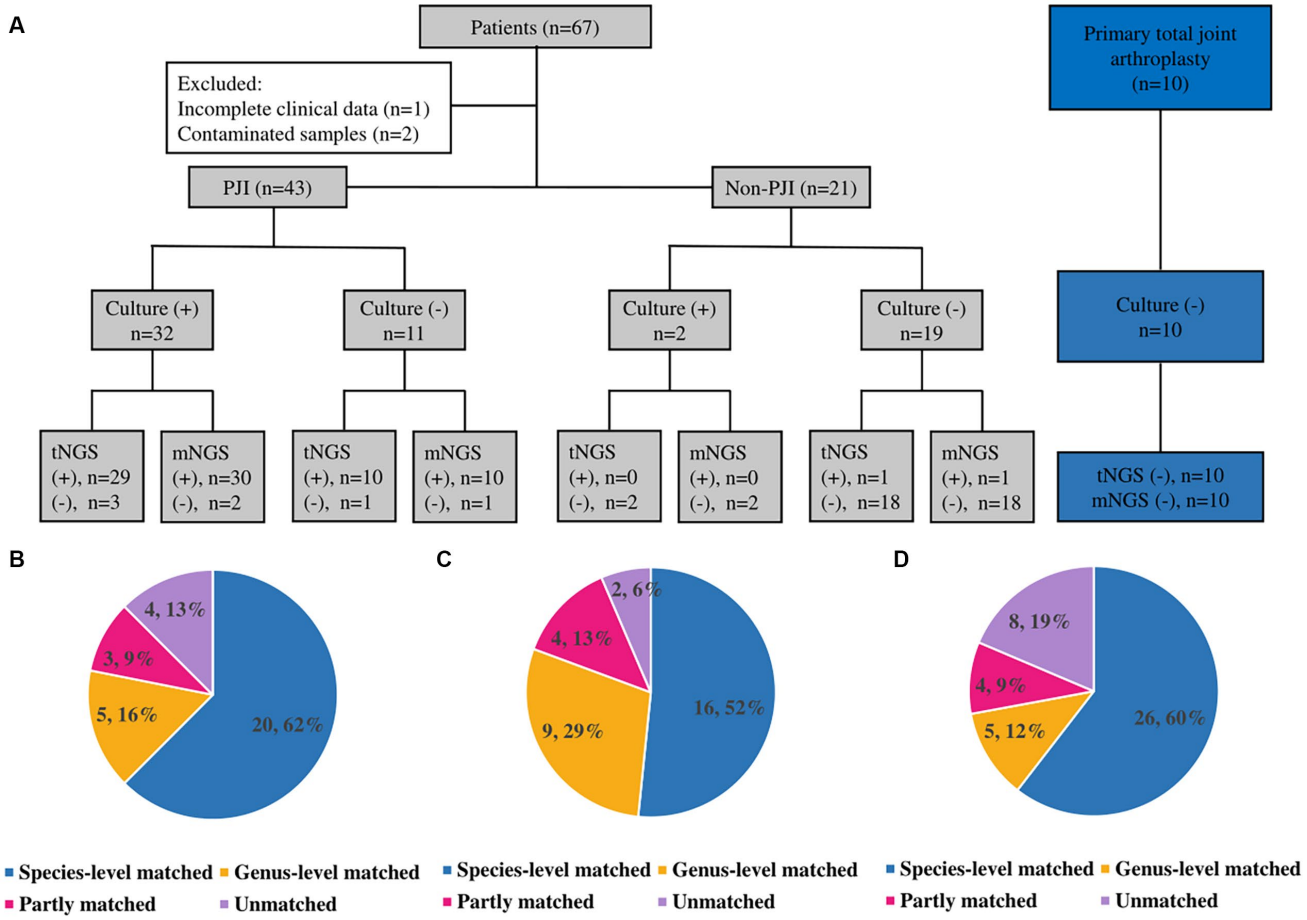
Comparison of turnaround time between mNGS and tNGS.

In this study, the cost per case was approximately $150 for tNGS and $260 for mNGS. mNGS is therefore 1.73 times more expensive than tNGS. We also compared the turnaround time for the two methods. tNGS and mNGS have roughly the same overall process, with tNGS requiring targeted amplification prior to library conduction, a process that takes approximately 3 h. mNGS takes more time for preprocessing, library production, and sequencing. In terms of total time, tNGS takes approximately 14.5 h per sample to generate interpretable results, while mNGS takes 28 h (Figure 2). Compared to mNGS, tNGS shortens the time by 13.5 h.

## Discussion

The identification of pathogenic microorganisms is key to the diagnosis and treatment of PJI, and it can guide the development of a rational antibiotic regimen, reduce drug resistance, and improve infection control (Tande and Patel, 2014). Our institution routinely uses a comprehensive culture strategy including multiple tissue cultures, sonication culture (Trampuz et al., 2007), optimized culture protocols (Fang et al., 2021), prolonged culture time (Schäfer et al., 2008), and improved tissue homogenization protocols prior to culture (Fang et al., 2021), yet microbial culture still had a negative rate of 25.6% (11/43) in this study. Here, the sensitivity of mNGS in the diagnosis of PJI was 93.0%, and the specificity was 95.2%. Potential pathogenic bacteria were detected in 90.9% of CN-PJI cases, which was similar to previous studies (Street et al., 2017; Thoendel et al., 2018; Huang et al., 2020a), which also showed the potential of nucleic acid detection technology for PJI. In this study, a novel culture independent pathogen detection technique was introduced, tNGS, to evaluate the diagnostic efficacy of tNGS for PJI and to compare it with mNGS and culture.

The advantage of tNGS with pre-defined targets is that the resources for detection can be focused on pre-defined targets. However, this also raises the concern of not being able to identify microorganisms outside the panel (Gaston et al., 2022). In our study, we selected specific sequences of 298 pathogens for the panels, which is probably the most pathogens incorporated into a panel in the field of PJI thus far, and this approach led to a sensitivity of 88.4% for tNGS in this study. In particular, tNGS detected 90.9% of the potentially pathogenic bacteria in CN-PJI, which shows the potential of tNGS for use in CN-PJI.

However, the detection sensitivity of mNGS in this study was still higher than that of tNGS (93.0% vs. 88.4%). One possible reason is that the samples used for the tNGS assay were not fresh samples but rather frozen samples, and prolonged freezing and rewarming may lead to degradation of DNA. Another reason is that *Streptococcus gallolyticus* and *Finegoldia magna*, which were detected by culture and mNGS, were not included in our tNGS panel. Rare pathogenic bacteria were left outside the panel, an unavoidable limitation of the targeting technique, which also occurs in multiplex PCR kits. Although some multiplex PCR kits have been shown to have good potential for PJI (Villa et al., 2017; Malandain et al., 2018), there are still concerns about the sensitivity of this approach. It is technically feasible to add new pathogens to these kits, but this requires substantial costs. Fortunately, the tNGS panel is reprogrammable, and incorporating new specific nucleic acid sequences of possible potential pathogens into the panel is not technically difficult and does not add excessive cost (Mertes et al., 2011).

The tNGS results matched the culture results at the species level better than the mNGS results matched the culture results (62% vs. 52%). A tremendous advantage of mNGS based on untargeted shotgun sequencing is the unbiased sampling of specimens, which allows mNGS to theoretically detect all pathogens, including viruses, bacteria, fungi, parasites, and undetected potential pathogens (Chiu and Miller, 2019). This also allows mNGS to detect a greater abundance of pathogens than tNGS. However, for PJI, the pathogenic microorganisms are essentially fungi and bacteria (Tande and Patel, 2014), and expending resources sequencing human-derived genes and detecting viruses and parasites increases the detection time, reduces the theoretical sensitivity, and increases costs (Chiu and Miller, 2019). In contrast, with the removal of interferences such as human-derived genes, the data volume of tNGS decreases dramatically, which makes tNGS superior to mNGS in terms of cost ($150 vs. $260) and turnaround time (14.5 h vs. 28 h), while maintaining good diagnostic efficacy, which is the advantage of targeted technologies.

For mNGS, typically less than 1% of reads are nonhuman, and the high host background in tissue samples results in a reduced number and proportion of pathogen reads (Chiu and Miller, 2019). Previous studies have found that the number of reads required for mNGS to detect pathogens in patients with PJI is much lower than that in patients with other infections, which may be related to the biased depletion of sequencing resources caused by the low concentration of planktonic pathogens and high relative human-derived nucleic acid concentrations in PJI (Trampuz et al., 2007; Huang et al., 2020a). This makes it difficult for mNGS to meet clinical requirements for the detection of antibiotic resistance. Here, we included drug resistance genes in the tNGS panel. In 37.5% of the CP-PJI cases, resistance genes were detected, and their resistance phenotypes were consistent with the culture results, with 75% being consistent. Clinically, the acquirement of resistance information by culture often takes several days, whereas tNGS results require only half a day, which allows physicians to obtain information to inform antibiotic regimen decisions more quickly. However, we must acknowledge that the ability of tNGS to detect resistance genes in PJI in this study was lower than we expected, and the low concentration of planktonic pathogens in PJI remains a possible cause; moreover, the resistance information obtained by tNGS has not been further validated, and future prospective studies are needed to confirm its accuracy.

There are some limitations of our study. 1. As mentioned earlier, the samples for tNGS were from biobank, while the data for tissue culture and mNGS were from fresh samples, which may have biased the results. Moreover, the samples used for the assay were not from consecutive cases, which may have resulted in selection bias. Future prospective studies need to be designed to improve the reliability of the study. 2. Our sample size is insufficient to determine the optimal threshold of contaminating bacteria for tNGS. Our interpretation of the tNGS results is based on our previously established criteria for mNGS. The interpretation of such results is arbitrary and subject to uncertainty. And, the microbial profile of knees versus hips in PJI potentially being different based on anatomic location, which is also a potential drawback to this study. 3. This was an exploratory study of the diagnosis of tNGS in PJI and no sample size calculations were performed prior to the study, and our sample size was limited. More samples or multicenter studies should be conducted to further validate the effectiveness of tNGS.

In summary, the advantage of tNGS over mNGS is that it excludes a range of interfering factors, including human-derived genes, and uses targeted technology to focus detection on pre-defined targets, which makes tNGS superior to mNGS in terms of cost and turnaround time. Moreover, the diagnostic efficacy of tNGS for PJI is not inferior to that of conventional diagnostic indices. The detection of drug resistance genes is one of the advantages of tNGS, which may be able to provide information on antibiotic resistance for PJI and guide the application of a more rational antibiotic regimen. However, tNGS cannot identify microorganisms outside the panel. But, the tNGS panel is reprogrammable, which allows tNGS to be readily improved. Finally, the potential for false positives exists with any microbial detection method, including culture, especially for the highly sensitive mNGS and tNGS. A multidisciplinary, multi-technology algorithm to diagnose PJI based on clinical characteristics is appropriate.

## Data availability statement

The datasets presented in this study can be found in online repositories. The names of the repository/repositories and accession number(s) can be found below: CNSA (http://db.cngb.org/cnsa/) of China National GeneBank Database (CNGBdb) with accession number CNP0001047.

## Ethics statement

The studies involving human participants were reviewed and approved by the Ethics Committee of First Affiliated Hospital of Fujian Medical of Fujian Medical University. The patients/participants provided their written informed consent to participate in this study.

## Author contributions

CH, YH, and ZW: writing of the manuscript and original draft preparation. XF and ZH: conceptualization. YH, XC, and YoL: methodology. YiL and ZW: software. CH and YH: validation and visualization. YC and WL: formal analysis. YC and XF: investigation. WZ and ZH: resources and funding acquisition. ZH, XC, and XF: data curation. WZ, XF, and ZH: writing – review and editing. XF and WZ: supervision. ZH: project administration. All authors contributed to the article and approved the submitted version.

## Funding

This work was supported by the National Natural Science Foundation Grant of China (grant number 82072458), the Foreign Cooperation Project of Science and Technology, Fujian Province, China (grant number 2019I0011), the Joint Funds for the Innovation of Science and Technology, Fujian Province, China (grant number 2019Y9301), and the Fujian Orthopaedic Bone and Joint Disease and Sports Rehabilitation Clinical Medical Research Center (grant number 2020Y2002).

## Conflict of interest

The authors declare that the research was conducted in the absence of any commercial or financial relationships that could be construed as a potential conflict of interest.

## Publisher’s note

All claims expressed in this article are solely those of the authors and do not necessarily represent those of their affiliated organizations, or those of the publisher, the editors and the reviewers. Any product that may be evaluated in this article, or claim that may be made by its manufacturer, is not guaranteed or endorsed by the publisher.
