# Supplementary material for: Multiplex PCR-based next generation sequencing as a novel, targeted and accurate molecular approach for periprosthetic joint infection diagnosis
Source: Front Microbiol. 2023 May 18;14:1181348. doi: 10.3389/fmicb.2023.1181348 (PMC10232910; doi:10.3389/fmicb.2023.1181348)
Supplement: Supplementary file 1 [file Data_Sheet_1.PDF]

Supplemental Material S1: Pathogens included in the PCR panel.

Remark		
F	fungus	
P	gram-positive bacterium	
N	gram-negative bacterium	
G	bacterial genus	
O	other	

NO.	Microorganisms	Sort
1	Trichosporon_asahii	F
2	Candida_albicans	F
3	Cladophialophora_bantiana	F
4	Mucor_irregularis	F
5	Fusarium_verticillioides	F
6	Coccidioides_immitis	F
7	Candida_dubliniensis	F
8	Candida_auris	F
9	Cryptococcus_gattii	F
10	Aspergillus_nidulans	F
11	Candida_glabrata	F
12	Fusarium_graminearum	F
13	Aspergillus_niger	F
14	Trichophyton_rubrum	F
15	Aspergillus_flavus	F
16	Penicillium_griseofulvum	F
17	Meyerozyma_guilliermondii	F
18	Aspergillus_parasiticus	F
19	Histoplasma_capsulatum	F
20	Fusarium_oxysporum	F
21	Wickerhamomyces_anomalus	F
22	Candida_parapsilosis	F
23	Penicillium_citrinum	F
24	Mucor_circinelloides	F
25	Cladophialophora_carrionii	F
26	Malassezia_furfur	F
27	Candida_krusei	F
28	Fusarium_poae	F
29	Fusarium	F
30	Trichosporon_ovoides	F
31	Papiliotrema_laurentii	F
32	Talaromyces_marneffei	F
33	Mucor	F
34	Rhizopus_oryzae	F
35	Fusarium_equiseti	F
36	Rhizomucor_endophyticus	F
37	Fonsecaea_pedrosoi	F
38	Exophiala_dermatitidis	F
39	Blastomyces_dermatitidis	F
40	Clavispora_lusitaniae	F
41	Coccidioides	F
42	Candida_tropicalis	F
43	Scedosporium	F
44	Fusarium_tricinctum	F
45	Lichtheimia_corymbifera	F
46	Sporothrix_schenckii	F

47	Magnusiomyces_capitatus	F
48	Aspergillus_terreus	F
49	Rhizomucor_pusillus	F
50	Candida_famata	F
51	Candida_haemulonis	F
52	Rhizopus_microsporus	F
53	Cryptococcus_neoformans	F
54	Aspergillus_fumigatus	F
55	Pneumocystis_jirovecii	F
56	Aspergillus_versicolor	F
57	Rhodotorula_graminis	F
58	Aspergillus_ochraceus	F
59	Mucor_racemosus	F
60	Aspergillus_clavatus	F
61	Aspergillus_oryzae	F
62	Fonsecaea_monophora	F
63	Fusarium_subglutinans	F
64	Trichophyton_benhamiae	F
65	Pasteurella	G
66	Sporothrix	G
67	Erysipelothrix	G
68	Brachyspira	G
69	Rhizomucor_sp	G
70	Alternaria	G
71	Actinomadura	G
72	Trichoderma	G
73	Stachybotrys	G
74	Serratia	G
75	Salmonella	G
76	Turicibacter	G
77	Leptotrichia	G
78	Microsporum	G
79	Cryptococcus	G
80	Verruconis_gallopava	G
81	Moraxella_osloensis	N
82	Bordetella_pertussis	N
83	Acinetobacter_baumannii	N
84	Burkholderia_mallei	N
85	Fluoribacter_bozemanae	N
86	Citrobacter_braakii	N
87	Pseudomonas_alcaligenes	N
88	Providencia_alcalifaciens	N
89	Klebsiella_aerogenes	N
90	Klebsiella_oxytoca	N
91	Chryseobacterium_indologenes	N
92	Pantoea_agglomerans	N
93	Edwardsiella_tarda	N
94	Bordetella_trematum	N
95	Vibrio_Vulnificus	N
96	Bacteroides_fragilis	N
97	Escherichia_coli	N
98	Pasteurella_multocida	N
99	Burkholderia_multivorans	N
100	Aggregatibacter_segnis	N

101	Pseudomonas_putida	N
102	Prevotella_bivia	N
103	Klebsiella_pneumoniae	N
104	Alcaligenes_faecalis	N
105	Hafnia_alvei	N
106	Citrobacter_freundii	N
107	Bartonella_bacilliformis	N
108	Shewanella_algae	N
109	Bartonella_henselae	N
110	Fusobacterium_necrophorum	N
111	Tropheryma_whipplei	N
112	Vibrio_cholerae	N
113	Corynebacterium_jeikeium	N
114	Ralstonia_mannitolilytica	N
115	Raoultella_ornithinolytica	N
116	Corynebacterium_urealyticum	N
117	Kingella_kingae	N
118	Moraxella_catarrhalis	N
119	Citrobacter_koseri	N
120	Campylobacter_jejuni	N
121	Burkholderia_pseudomallei	N
122	Neisseria_gonorrhoeae	N
123	Haemophilus_influenzae	N
124	Ewingella_americana	N
125	Morganella_morganii	N
126	Elizabethkingia_meningoseptica	N
127	Neisseria_meningitidis	N
128	Streptobacillus_moniliformis	N
129	Ralstonia_pickettii	N
130	Proteus_vulgaris	N
131	Pseudomonas_oryzihabitans	N
132	Proteus_mirabilis	N
133	Moraxella_lacunata	N
134	Eikenella_corrodens	N
135	Capnocytophaga_canimorsus	N
136	Ochrobactrum_anthropi	N
137	Cardiobacterium_hominis	N
138	Pluralibacter_gergoviae	N
139	Vibrio_alginolyticus	N
140	Salmonella_Typhi	N
141	Legionella_pneumophila	N
142	Stenotrophomonas_maltophilia	N
143	Aeromonas_hydrophila	N
144	Yersinia_pestis	N
145	Providencia_stuartii	N
146	Shigella_sonnei	N
147	Campylobacter_fetus	N
148	Burkholderia_gladioli	N
149	Pseudomonas_aeruginosa	N
150	Bartonella_vinsonii	N
151	Yersinia_enterocolitica	N
152	Porphyromonas_gingivalis	N
153	Burkholderia_cepacia	N
154	Serratia_liquefaciens	N

155	Bartonella_elizabethae	N
156	Acinetobacter_calcoaceticus	N
157	Enterobacter_cloacae	N
158	Serratia_marcescens	N
159	Brucella	N
160	Dialister_pneumosintes	N
161	Elizabethkingia_miricola	N
162	Klebsiella_variicola	N
163	Ehrlichia	O
164	Coxiella_burnetii	O
165	Ehrlichia_Chaffeensis	O
166	Chlamydia_pneumoniae	O
167	Mycoplasma_pneumoniae	O
168	Ureaplasma_urealyticum	O
169	Coxiella	O
170	Rickettsia_felis	O
171	Chlamydia_trachomatis	O
172	Mycoplasma_genitalium	O
173	Anaplasma_phagocytophilum	O
174	Borrelia	O
175	Neorickettsia_sennetsu	O
176	Orientia_tsutsugamushi	O
177	Chlamydia_psittaci	O
178	Rickettsia_prowazekii	O
179	Staphylococcus_arlettae	P
180	Nocardia_brasiliensis	P
181	Corynebacterium_diphtheriae	P
182	Actinomadura_pelletieri	P
183	Clostridium_septicum	P
184	Mycobacterium_intracellulare	P
185	Staphylococcus_epidermidis	P
186	Mycobacterium_xenopi	P
187	Clostridium_perfringens	P
188	Staphylococcus_chromogenes	P
189	Mycobacterium_smegmatis	P
190	Cutibacterium_acnes	P
191	Listeria_monocytogenes	P
192	Streptococcus_downei	P
193	Nocardia_brevicatena	P
194	Staphylococcus_carnosus	P
195	Mycobacterium_africanum	P
196	Streptococcus_pneumoniae	P
197	Enterococcus_faecalis	P
198	Staphylococcus_saprophyticus	P
199	Nocardia_cyriacigeorgica	P
200	Mycobacterium_gordonae	P
201	Mycobacterium_colombiense	P
202	Mycobacterium_chelonae	P
203	Mycobacterium_marinum	P
204	Staphylococcus_lentus	P
205	Streptococcus_mitis	P
206	Staphylococcus_gallinarum	P
207	Streptococcus_cristatus	P
208	Staphylococcus_pseudintermedius	P

209	Clostridioides_difficile	P
210	Mycobacterium_tuberculosis_complex	P
211	Streptococcus_gallolyticus	P
212	Staphylococcus_aureus	P
213	Mycobacterium_kansasii	P
214	Staphylococcus_cohnii	P
215	Staphylococcus_kloosii	P
216	Mycobacterium_ulcerans	P
217	Bacillus_cereus	P
218	Staphylococcus_lugdunensis	P
219	Mycobacterium_scrofulaceum	P
220	Mycobacterium_leprae	P
221	Gemella_morbillorum	P
222	Streptococcus_equi	P
223	Mycobacterium_massiliense	P
224	Staphylococcus_equorum	P
225	Corynebacterium_macginleyi	P
226	Streptococcus_milleri	P
227	Staphylococcus_simulans	P
228	Staphylococcus_xylosus	P
229	Streptococcus_pyogenes	P
230	Enterococcus_avium	P
231	Mycobacterium_avium	P
232	Actinomyces_bovis	P
233	Mycobacterium_bovis	P
234	Streptococcus_bovis	P
235	Mycobacterium_abscessus	P
236	Nocardia_abscessus	P
237	Clostridium_novyi	P
238	Mycobacterium_fortuitum	P
239	Nocardia_farcinica	P
240	Granulicatella_adiacens	P
241	Clostridium_tetani	P
242	Enterococcus_casseliflavus	P
243	Mycobacterium_gilvum	P
244	Actinomyces_odontolyticus	P
245	Streptococcus_canis	P
246	Abiotrophia_defectiva	P
247	Mycobacterium_tuberculosis	P
248	Staphylococcus_hominis	P
249	Mycoplasma_hominis	P
250	Mycobacterium_genavense	P
251	Staphylococcus_haemolyticus	P
252	Clostridium_histolyticum	P
253	Clostridium_botulinum	P
254	Staphylococcus_caprae	P
255	Staphylococcus_schleiferi	P
256	Enterococcus_faecium	P
257	Staphylococcus_sciuri	P
258	Bacillus_anthracis	P
259	Micrococcus_luteus	P
260	Streptococcus_dysgalactiae	P
261	Staphylococcus_capitis	P
262	Nocardia_otitidiscaviarum	P

263	Corynebacterium_striatum	P
264	Staphylococcus_warneri	P
265	Streptococcus_agalactiae	P
266	Staphylococcus_vitulinus	P
267	Nocardia_nova	P
268	Nocardia_asteroides	P
269	Streptococcus_constellatus	P
270	Mycobacterium_kumamotonense	P
271	Streptococcus_anginosus	P
272	Peptostreptococcus_anaerobius	P
273	Streptococcus_ovis	P
274	Actinomyces_israelii	P
275	Streptococcus_infantarius	P
276	Streptococcus_intermedius	P
277	Staphylococcus_intermedius	P
278	Streptococcus_hyointestinalis	P
279	Streptococcus_suis	P
280	Rhodococcus_rhodochrous	P
281	Kocuria_rosea	P
282	Rothia_mucilaginosa	P
283	Staphylococcus_hyicus	P
284	Streptococcus_equinus	P
285	Enterococcus_gallinarum	P
286	Veillonella_parvula	P
287	Mycobacterium_asiaticum	P
288	Mycobacterium_bohemicum	P
289	Mycobacterium_canettii	P
290	Mycobacterium_celatum	P
291	Mycobacterium_haemophilum	P
292	Mycobacterium_malmoense	P
293	Mycobacterium_simiae	P
294	Mycobacterium_szulgai	P
295	Mycobacterium_triplex	P
296	Mycobacterium_bacteremicum	P
297	Mycobacterium_mucogenicum	P
298	Mycobacterium_neoaurum	P

Supplemental Material S2. Drug resistance genes and drug resistance phenotypes included in the PCR panel.

Resistance phenotypes	Resistance genes
methicillin/beta-lactam	mecA/mecB/mecC
carbapenems/penicillins/cephalosporins	CcrA/FEZ-1/GOB-1/IND/KPC/NMC/OXA-181/OXA-198/OXA-23/OXA-24/OXA-48/OXA-69/OXA-51/OXA-55/SFH-1/SPM-1/VIM
penicillins/cephalosporins Can be inhibited by β-	CTX-M-1/CTX-M-2/CTX-M-25/GES/OXA-1/OXA-2/OXA-7/OXA-9/OXA-10/PER/ROB-1/SHV/TEM/VEB
penicillins/cephalosporins Not inhibited by β-	ACC/ACT/ADC/AmpC/CMY/DHA/MIR

macrolides/Lincosamides/Streptomycin	ereA/ereB/ermB/mefA/mefE/msrA/macA/mphB
tetracyclines	EMR/tetB/tetC/tetD/tetE/tetK/tetL/tetM/tetO/tetQ
aminoglycosides	AAC(2')/AAC(6')/AAC(3)/ANT/APH/ARM/RMT
polypeptide	vanA/vanB/vanC/vanD/vanE/vanG/tetS/tetT/tetW
sulfonamides	DFR/SUL
quinolones	QAC/qnrA
Chloramphenicols	CAT/cmlA/floR
colistin	mcr-1
multi-drug resistant	ACR/ADE/tolC
